# Serratus anterior plane block for acute pain management after pectus excavatum repair

**DOI:** 10.3389/fsurg.2023.1305326

**Published:** 2024-01-08

**Authors:** Gongmin Rim, Hyung Joo Park, Seungyoun Kang, Jin Yong Jeong, Jungmin Koo, Il-Tae Jang, Saemi Bae

**Affiliations:** ^1^ Department of Thoracic and Cardiovascular Surgery, Gangnam Nanoori Hospital, Seoul, Republic of Korea; ^2^ Department of Thoracic and Cardiovascular Surgery, Incheon St. Mary's Hospital, College of Medicine, The Catholic University of Korea, Incheon, Republic of Korea; ^3^ Seoul St. Mary's Hospital, The Catholic University of Korea, Seoul, Republic of Korea

**Keywords:** minimally invasive repair of pectus excavatum, pain control, pectus excavatum, serratus anterior plane block, cryoanalgesia

## Abstract

**Introduction:**

Conventional postoperative pain management using an intravenous (IV) patient-controlled approach or thoracic epidural analgesia is suboptimal following minimally invasive repair of the pectus excavatum (MIRPE). Recently, cryoanalgesia has gained popularity owing to its superior pain control outcomes compared to those associated with conventional methods. However, because of its invasiveness, additional instrumentation requirement, and limited effect at early postoperative periods, we hypothesized that serratus anterior plane block (SAPB) could be an effective method for post-repair pain management and a possibly superior alternative.

**Methods:**

We conducted a retrospective cohort study of pediatric patients who had undergone MIRPE between March 2022 and August 2023. We compared the efficacy of pain control in three groups among 74 patients: Group *N* (conventional pain management, *n* = 24), Group C (cryoanalgesia, *n* = 24), and Group S (SAPB, *n* = 26). Group *N* received IV patient-controlled analgesia (PCA) and a subcutaneous local anesthetic infusion. Group C received bilateral cryoanalgesia on the fourth and seventh intercostal nerves using a cryoprobe at −80°C for 2 min during the operation and IV-PCA postoperatively. Group S received continuous bilateral SAPB with 0.25% ropivacaine and IV-PCA. The pain levels were measured using the visual analog scale (VAS; resting and dynamic), and the total IV rescue analgesic consumption was determined.

**Results:**

The three groups had similar baseline characteristics. Group S showed significantly less pain throughout the immediate postoperative course, resting VAS score at 3 h (Group *N*, 7.21 vs. Group C, 5.75 vs. Group S, 3.81; *p* < 0.001), and prominent less total IV rescue analgesic consumption (Group *N*, 116.16 mg vs. Group C, 52.75 mg vs. Group S, 16.61 mg; *p* < 0.001).

**Conclusion:**

SAPB resulted in better postoperative pain control than that associated with cryoanalgesia and conventional pain management after pectus excavatum repair, As it was effective in the immediate postoperative period, achieving a VAS score of <4 points (moderate pain) at 3 h postoperatively, it may play an important role and replace invasive cryoanalgesia in the management of pain after pectus surgery.

## Introduction

Advancements in minimally invasive surgical repair of the pectus excavatum (PE) in the last two decades have enabled significantly improved patient outcomes by developing better surgical techniques and instrumentation (1, 2). However, postoperative pain caused by major chest wall remodeling remains a challenge and optimal solutions are lacking. When elevating the depressed chest wall by the pectus bar(s), constant and excessive pressure is placed on the rib cage, triggering intense postoperative pain (3, 4). Conventional postoperative pain management for the PE repair includes intravenous (IV) patient-controlled analgesia (PCA), thoracic epidural analgesia, and continuous local anesthetic infusion (ON-Q PainBuster; B Braun, Hessen, Germany), all of which have limitations in optimal pain management.

Although earlier data have suggested that thoracic epidural analgesia offers maximum benefit in this setting (5–7), new evidence for equal efficacy of IV-PCA has emerged (8, 9). Ultimately, both approaches are suboptimal solutions for post-repair pain, each presenting its own disadvantages. A major concern in thoracic epidural analgesia is the development of catheter-related complications, ranging from minor issues (dislodgement or kinking) to severe problems (neurologic damage). Using opioids for IV-PCA is a problem because it may induce opioid-related side effects, such as nausea, vomiting, and even respiratory depression (9). Cryoanalgesia has recently gained popularity owing to its superior pain control outcomes compared to those associated with conventional methods (10–12). However, cryoanalgesia requires a thoracoscopic approach, single-lung ventilation, intrathoracic procedures, and additional instrumentation, which are invasive and require more time and cost (13). As a better alternative, we adopted serratus anterior (SA) plane block (SAPB), a technique involving local anesthetic infusion through an extrathoracic catheter (14, 15). Therefore, we hypothesized that SAPB would be a superior method for pain management compared to that associated with conventional treatment and cryoanalgesia to help alleviate immediate postoperative pain and reduce opioid use after minimally invasive PE repair.

## Materials and methods

### Study design

This retrospective analysis enrolled 74 patients out of 126 patients who underwent pectus excavatum (PE) repair surgery between March 2022 and August 2023, excluding those who met the following exclusion criteria. (1) Patients with a history of prior pectus excavatum repair resulting in recurrence. (2) Patients with a history of chronic pain or psychological disorders. (3) Patients with incomplete medical records, particularly with regard to pain scores. Notably, Group C and Goup N's subset of patients were published previously (13).

All pectus repairs were performed by the corresponding author (H.J.P). Demographic data, medications administered, surgical and medical histories, and perioperative data [i.e., operative time, pain level, opioid use, complications, and length of hospital stay (LOS)] were collected through patient interviews and electronic medical records. The total IV rescue analgesic consumption was determined using approximate morphine milligram equivalent (MME) (16, 17). The patients were divided into groups according to the pain control modality as follows: Group *N*, conventional pain management (*n* = 24); Group C, cryoanalgesia (*n* = 24); and Group S, SAPB (*n* = 26) for the evaluation and comparison of efficacy and adverse reactions.

**Figure 1:**
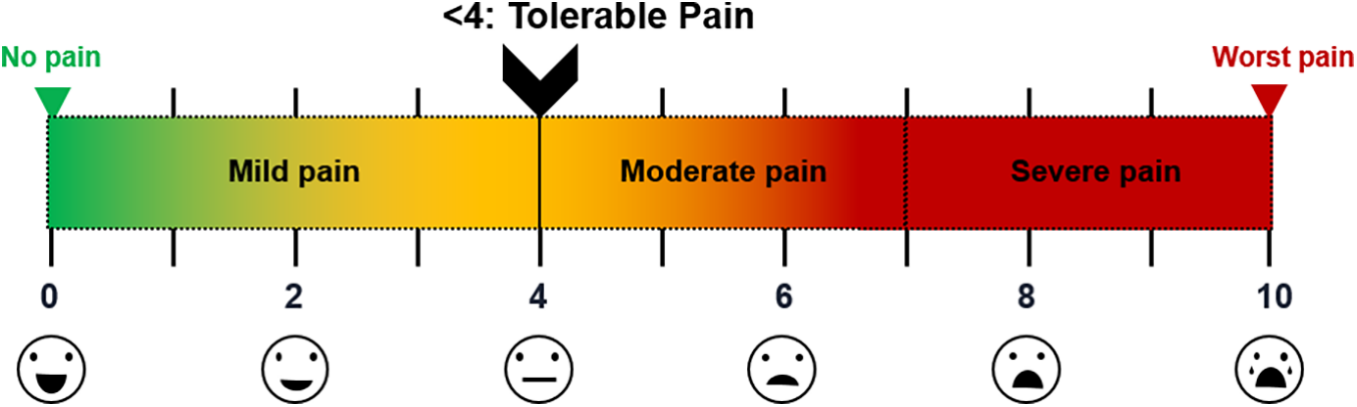
VAS scores of postoperative pain Illustration of pain levels; scores <4 points viewed as tolerable (mild pain). VAS, visual analog scale.

The severity of postoperative pain was determined by the designated investigator using a visual analog scale (VAS) at various intervals (1, 3, 6, 12, 24, 48, and 72 h) postoperatively. Patients were instructed to score their pain levels on a scale from 0 (no pain) to 10 (worst pain) points (Figure 1) in both the resting (VAS-R) and dynamic (VAS-D) states, VAS-R in the supine position, and VAS-D in the upright position when coughing. VAS scores <4 indicated tolerable pain control state (Figure 1). The postoperative pain scale was assessed by an independent registered nurse.

We measured the total IV rescue analgesic consumption at postoperative intervals (6, 12, 24, 48, and 72 h), LOS, complications (pneumothorax, wound complications, reoperation, pectus bar dislocation, bleeding necessitating transfusion, and neurological or cardiac issues), total operative time, and block time of cryoanalgesia or SAPB.

### Surgical procedures

All pectus repair procedures were performed by a single surgeon at a single center and the surgical techniques were identical, except for the nerve block procedure. Patients were positioned supine with both arms freely suspended in overhead slings to avoid arm stretching. Bilateral 1.5-cm skin incisions were made at the midaxillary line, forming pockets at the subcutaneous layer.

The principal operative technique included total craned lifting of the sternum, pectoscope (PrimeMed, Seoul, Republic of Korea) visualization/dissection, multiple bar placement with bridge plate fixation (PrimeMed), and flarebuster/magic string technique using No. 5 Ethibond strings (Ethicon Inc., Somerville, NJ, USA) (18, 19).

### Crane-powered remodeling of the entire chest wall

Before repair, the depressed sternum was fully elevated to ensure that the pectus bars were accurately positioned without any effort or risk of injury to other organs (20). Sternal pre-lifting was made using the Easy crane system (PrimeMed) over the level of target chest wall height in all cases. By doing this, the chest wall depression was elevated with crane power, eliminating the need for pectus bar turnover power. Notably, the crane was set up with sternal wiring (1) or sternal screws, which is our novel system (21, 22).

Our surgical policy for correcting deformities focused on remodeling the entire chest wall; not just raising the depressed portion of the wall but also covering the entire anterior chest wall between both anterior axillary lines to achieve anatomic integrity of the transformed chest wall.

### Cryoanalgesia

**Figure 2:**
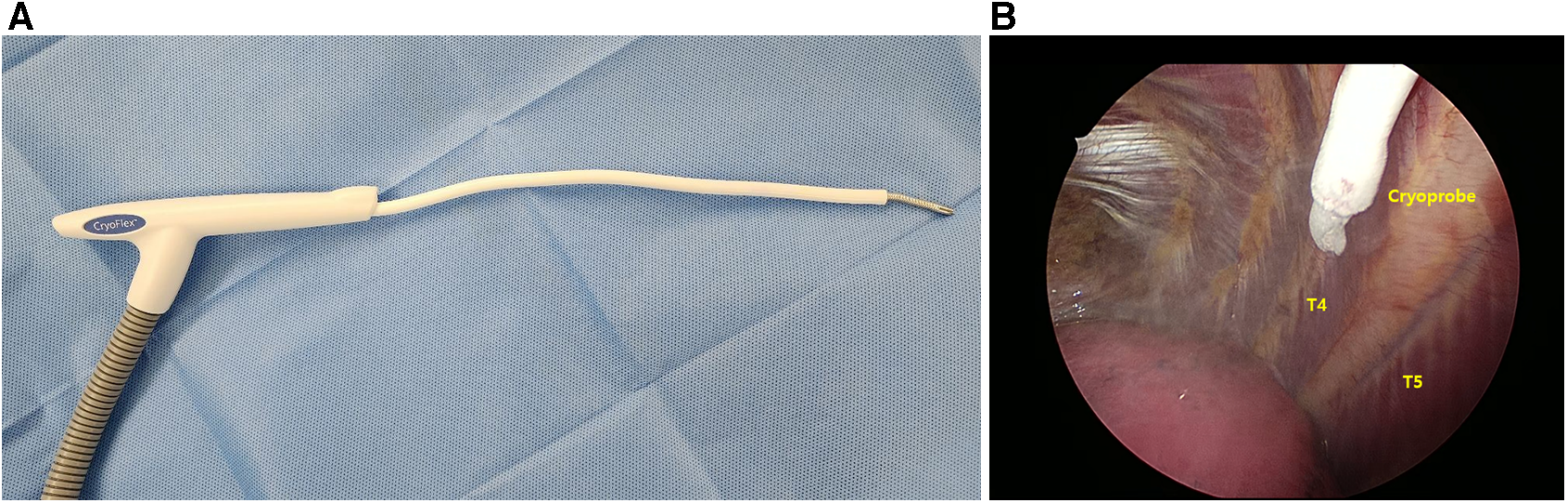
Intraoperative cryoanalgesia. (**A**) Cardioblate cryoFlex probe (Medtronic, Inc) with malleable tip; and (**B**) thoracoscope-assisted cryoablation of T5, right side (T4–T7 treated at −80°C for 2 min bilaterally).

Cryoanalgesia (intercostal nerve cryoablation) was administered before PE repair. A double-lumen endotracheal tube was used for selective ventilation to avoid lung injury and to maintain a clear view of the target intercostal nerves during the ablation period. A Cardioblate CryoFlex Surgical Ablation Console (Medtronic Inc., Minneapolis, MN, USA) was used (Figure 2A). The cryoprobe was applied to the fourth to seventh intercostal nerves (T4–T7) bilaterally under video-thoracoscopic assistance and cooled to −80°C using Argon gas for 2 min (Figure 2B).

### SAPB

**Figure 3:**
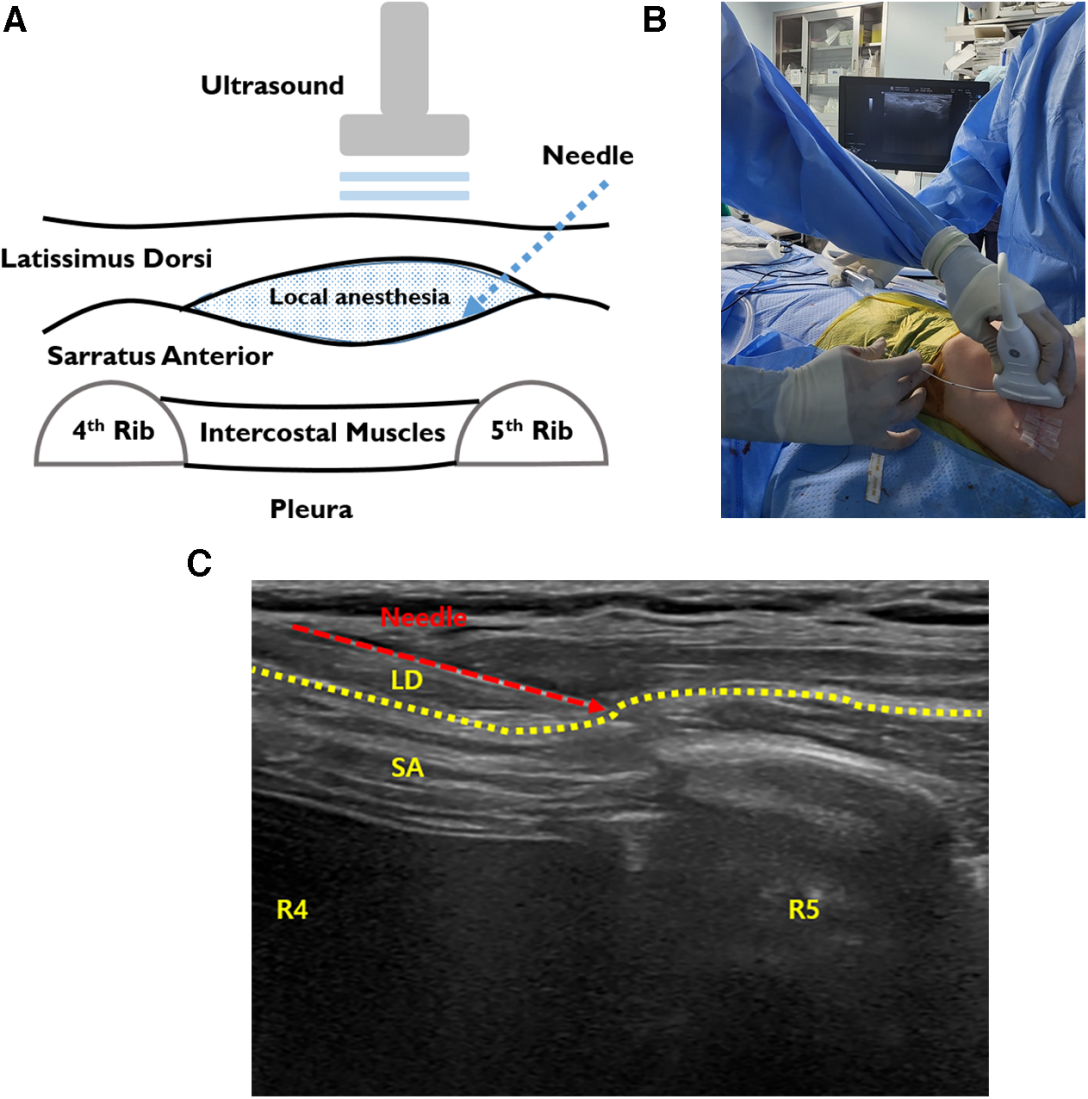
Intraoperative SAPB. (**A**) Illustration of SAPB, injecting local anesthetic agents between the SA and LD. (**B**) The procedure of ultrasound-guided SAPB before repair. (**C**) Bilateral ultrasound-guided painfusor catheter placement for continuous SAPB after PE repair. LD, latissimus dorsi; SA, serratus anterior; SAPB, serratus anterior plane block.

SAPB was performed before PE repair. After the patient was in the supine position, a high-frequency linear ultrasound transducer was placed anterior to the midaxillary line at the level of the fourth or fifth ribs on the side of the block (Figure 3A) (23–25). After identification of the SA and latissimus dorsi (LD), the block needle (20 gauge -BD Perisafe™ Modified Tuohy Point Epidural Needles, BD Inc., Eschborn, Germany) was advanced between the interfacial plane of SA and LD using ultrasound in-plane technique. First, 5–10 ml of saline was injected to open the interfacial space between the SA and LD and, then, 30 mL of 0.25% ropivacaine (maximum dose of ropivacaine: 3 mg/kg) with 1:200000 epinephrine and adjuvants of 5 mg dexamethasone and 50 mcg Fentanyl to increase the quality and efficacy of local anesthecis under sono guide bilaterally was injected bilaterally (Figure 3B). It blocks the lateral cutaneous branch of the 2nd–9th intercostal nerves and the long thoracic nerve that covers the area of the anterior and lateral chest walls. After PE repair, for continuous SAPB, infusion catheters (Painfusion, Baxter Inc., Deerfield, IL, USA) were placed in the SA planes on both sides under ultrasound-guide. The elastomeric infusion pump was connected to the catheter, and 0.3% ropicavaine was delivered in a flow of 5 ml/h continuously (Figure 3C).

### Basal pain management protocol

All study participants received a standardized pain regimen according to the institutional protocol. General anesthesia was induced using IV lidocaine (1–2 mg/kg; maximum, 100 mg) and propofol (2–4 mg/kg; maximum, 150 mg), with rocuronium (0.6–1 mg/kg; maximum, 60 mg) or vecuronium (0.1 mg/kg; maximum, 10 mg) to facilitate endotracheal intubation. Moreover, 1.2–1.5% sevoflurane was also used for maintenance in a mixture of 50% air and 50% O_2_ with a bispectral index less than 60.

The conventional postoperative pain management protocol includes: (1) IV-PCA, (2) subcutaneous local anesthetic infusion, (3) shots of nonsteroidal analgesics on demand, and (4) oral basal analgesics.

IV-PCA was initiated at the recovery room in all patients in the three groups using pumps delivering fentanyl (15 mcg/kg) in 100-ml normal saline (basal rate, 0.5 ml/h; bolus dose, 1 ml; lockout time, 10 min). The orally administered postoperative analgesics for both groups were ibuprofen (10–15 mg/kg, every 6 h) and acetaminophen (10–15 mg/kg, every 6 h). For breakthrough pain, ketorolac (0.5 mg/kg, every 6 h) and pethidine (0.5 mg/kg, every 6 h) were administered as IV rescue analgesics on an as-needed basis. IV rescue analgesia was administered based on the patient's request for relief from breakthrough pain, without any direct involvement from the investigator. The total rescue analgesic consumption was determined by conversion to oral MMEs. Patients with postoperative emesis or nausea received ondansetron (0.1–0.15 mg/kg; maximum, 4 mg).

For subcutaneous infusion of local anesthetic agents (ON-Q PainBuster), catheters (7.5 or 15 cm) were placed bilaterally at the posterior axillary lines after repair. A 240-ml reservoir released 0.3% ropivacaine at a fixed rate of 5 ml/h. The catheters were removed at 2–3 days postoperatively.

Patients in Group C received video thoracoscopy-assisted bilateral cryoanalgesia (T4–T7), besides the conventional pain management protocol. Patients in Group S received bilateral SAPB and a continuous local anesthetic catheter using a conventional pain management protocol.

### Statistical analysis

Normally distributed continuous data, skewed data, and categorical data are presented as means [standard deviation (SD)] values, medians (interquartile ranges), and numbers, respectively. Descriptive statistics (number of individuals, mean, SD, median, minimum, maximum, and quartile range) and categorical data were conveyed as subject numbers and percentages, checked for normal distribution and relations, and assessed using analysis of variance or the Kruskal–Wallis test. All computations were performed using standard software (SPSS v. 25.0; IBM Corp., Armonk, NY, USA).

## Results

Among the 74 enrolled patients, there were no group differences in age, height, weight, body mass index, sex, American Society of Anesthesiologists class, or PE type. The baseline characteristics of the study groups are presented in Table 1.

**Table 1:** Baseline characteristics of study groups.

	Conventional (group *N*) (*n* = 24)	Cryo (group C) (*n* = 24)	SAPB (group S) (*n* = 26)	*p* value*
Age, years	14.92 (2.83)	14.46 (2.65)	14.31 (2.35)	0.696
Height, cm	166.85 (10.47)	167.73 (10.34)	166.35 (11.26)	0.816
Weight, kg	49.53 (10.82)	50.49 (11.14)	50.17 (8.94)	0.948
BMI, kg/m^2^	17.61 (2.43)	17.81 (2.59)	17.87 (1.62)	0.917
Male sex (percentage)	20 (83.33)	21 (87.5)	21 (80.77)	0.816
Race, Asian	24 (100)	24 (100)	26 (100)	1.000
ASA class				0.064
ASA Ⅰ	22 (91.66)	24 (100)	25 (96.2)	
ASA Ⅱ	2 (8.33)		1 (3.8)	
PE symmetric type	13 (54.17)	13 (54.17)	16 (61.54)	0.549

Cryo, cryoanalgesia; ASA, American society of anesthesiologists; BMI, body mass index; SD, standard deviation.

Data expressed as mean (SD) or number (%).

* Significance, *p *< 0.05.

Perioperative clinical and radiologic characteristics are summarized in Table 2.

**Table 2:** Perioperative characteristics of study groups.

	Conventional (group *N*) (*n* = 24)	Cryo (group C) (*n* = 24)	SAPB (group S) (*n* = 26)	*p* value*
Depression index (DI)				
Pre (range)	1.66 (1.22–2.50, 0.31)	1.46 (1.22–2.39, 0.25)	1.62 (1.32–2.56, 0.28)	0.226
Post (range)	1.04 (1.00–1.22, 0.05)	1.04 (1.00–1.15, 0.04)	1.02 (1.00–1.07, 0.02)	0.004
Δ DI	0.62 (0.17–1.42, 0.31)	0.42 (0.19–1.27, 0.23)	0.60 (0.26–1.56, 0.28)	0.570
Haller index (HI)				
Pre	4.58 (3.29–8.70)	4.60 (3.13–11.37)	4.04 (3.15–6.15) 2.41 (2.01–3.08, 0.24)	0.023
Post	2.65 (2.24–3.05, 0.20)	2.55 (2.11–3.41, 0.30)		0.004
Δ HI	1.93 (0.67–5.72, 1.23)	2.05 (0.54–8.94, 1.68)	1.63 (0.4–3.70, 0.89)	0.500
Operative time, min	124.86 (70–155)	157.86 (100–199)	110.96 (75–180)	0.001
Block time, min		30.33 (20–45, 7.82)	17.77 (14–30, 3.39)	<0.001
Pectus bar size, in	13.38 (11–15, 1.10)	13.13 (11–15, 1.03)	13.19 (11–15, 1.01)	0.682
Number of pectus bars				0.092
1	0	0	0	
2	3 (12.5)	6 (25)	1 (3.8)	
3	21 (87.5)	18 (75)	25 (96.2)	
Pectus bar shape				
Parallel	5 (20.83)	2 (8.33)	3 (11.54)	0.993
Cross	3 (12.50)	3 (12.50)	0 (0)	0.061
XI	16 (66.67)	19 (79.17)	23 (88.46)	0.316
Crane application	24 (100)	24 (100)	26 (100)	1.000
Pectoscope	24 (100)	24 (100)	26 (100)	1.000
Flare buster	24 (100)	24 (100)	26 (100)	1.000
Magic string	24 (100)	24 (100)	26 (100)	1.000

Cryo, cryoanalgesia; SD, standard deviation.

Data expressed as mean (range, SD) or number (%).

* Significance, *p *< 0.05.

All groups underwent surgical PE repair under crane application using a pectoscope, multiple pectus bars (parallel, cross, or XI shaped), and the flarebuster/magic string technique. The pectus bar size and number of pectus bars did not differ significantly between the groups. However, there were no single-bar repairs in any group.

**Figure 4:**
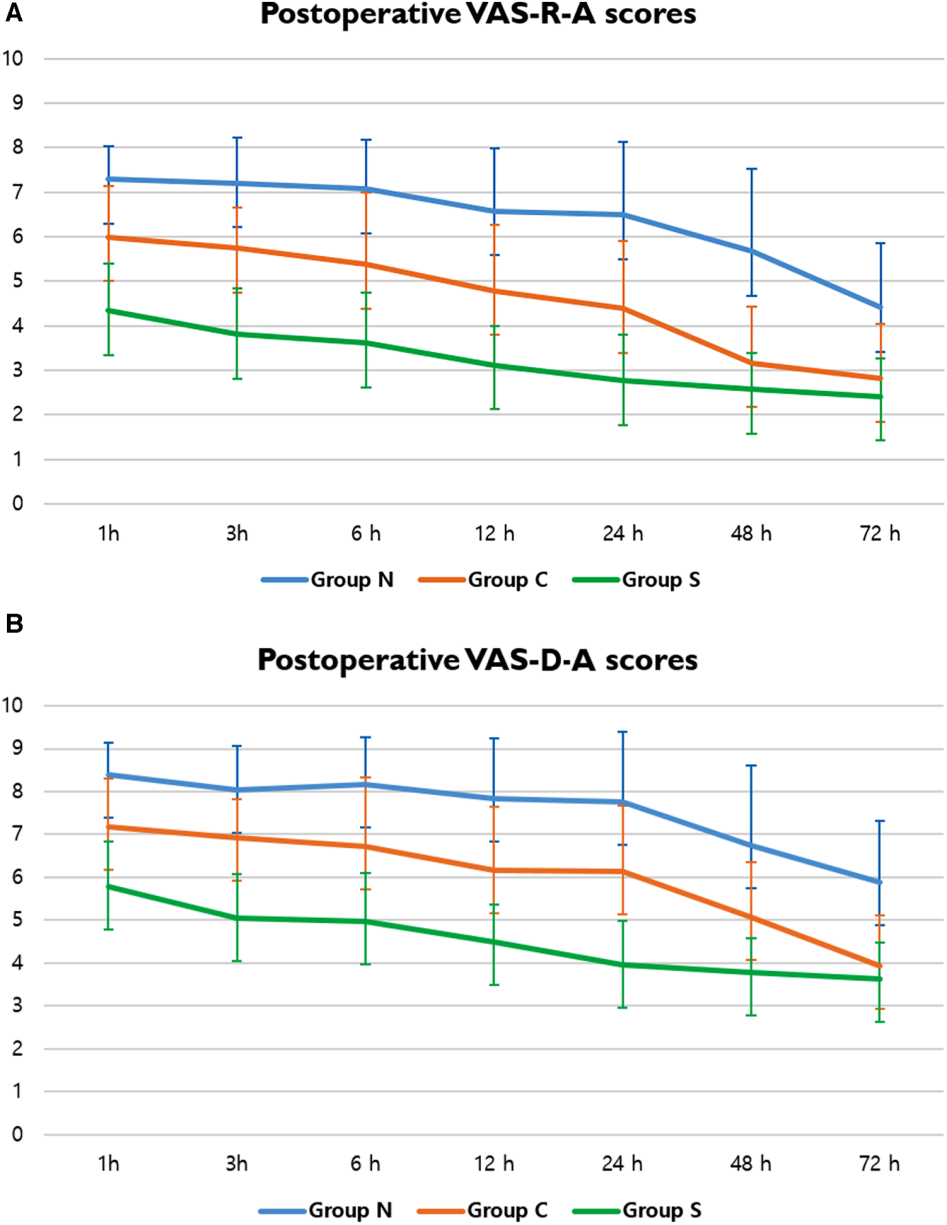
VAS scores of postoperative pain. (**A**) Postoperative VAS-R-A scores in Groups N, C, and S; and (**B**) postoperative VAS-D-A scores in Groups N, C, and S. A, anterior chest wall; VAS, visual analog scale; VAS-D, dynamic VAS score; VAS-R, resting VAS score.

Pain score assessments, whether resting (VAS-R) or dynamic (VAS-D), were performed at the anterior (VAS-R-A, VAS-D-A) and lateral (VAS-R-L, VAS-D-L) chest walls (Tables 3, 4). The mean VAS score was significantly lower in Group S for the entire 72-h postoperative period, with resting scores (VAS-R-A, VAS-R-L) being <4 points for the 3 h (3.81 points) and 24-h (3.57 points) periods, respectively. However, the VAS-R-A and VAS-R-L scores for Group *N* were >4 points during the full 72-h postoperative period, and the resting scores (VAS-R-A, VAS-R-L) were <4 points at 48 h (3.17 points) and 24 h (3.61 points) (Figure 4A). In Group S, the dynamic scores were <4 points at 24 h, respectively, whereas the VAS-D were >4 points during the entire postoperative period in Group *N*. The dynamic scores were <4 points at postoperative 72 h in Group C (Figure 4B).

**Table 3:** Postoperative resting VAS (VAS-R) scoring, shown by a group.

	Conventional (group *N*) (*n* = 24)	Cryo (group C) (*n* = 24)	SAPB (group S) (*n* = 26)	*p* value*
VAS-R-A				
1h	7.29 (6–9, 0.751)	6.00 (4–8, 1.142)	4.35 (2–6, 1.056)	<0.001
3h	7.21 (5–9, 1.021)	5.75 (4–7, 0.897)	3.81 (2–6, 1.021)	<0.001
6 h	7.08 (5–10, 1.10)	5.38 (2–8, 1.61)	3.62 (1–6, 1.134)	<0.001
12 h	6.58 (3–9, 1.41)	4.79 (2–7, 1.47)	3.12 (1–5, 0.864)	<0.001
24 h	6.50 (4–10, 1.64)	4.38 (2–8, 1.53)	2.77 (1–6, 1.032)	<0.001
48 h	5.67 (2–10, 1.86)	3.17 (1–6, 1.27)	2.58 (2–6, 0.809)	<0.001
72 h	4.42 (2–7, 1.44)	2.83 (1–6, 1.20)	2.42 (1–5, 0.857)	0.003
VAS-R-L				
1 h	6.95 (4–9, 1.32)	5.71 (4–8, 1.16)	4.19 (2–6, 1.06)	<0.001
3 h	7.05 (5–9, 0.95)	5.50 (4–7, 0.88)	3.57 (2–7, 1.14)	<0.001
6 h	6.25 (4–10, 1.67)	4.71 (2–8, 1.71)	3.65 (1–7, 1.47)	0.003
12 h	5.96 (3–9, 1.49)	3.92 (2–7, 1.47)	3.08 (1–5, 0.89)	<0.001
24 h	5.79 (3–10, 1.74)	3.63 (1–7, 1.44)	2.69 (1–6, 1.22)	<0.001
48 h	5.08 (2–10, 1.84)	2.75 (1–5, 1.11)	2.58 (1–8, 1.42)	<0.001
72 h	4.04 (2–7, 1.52)	2.54 (1–5, 1.02)	2.27 (1–4, 0.96)	0.003

SD, standard deviation; Cryo, cryoanalgesia; VAS, visual analog scale; VAS-R-A, resting VAS score at anterior chest wall; VAS-R-L, resting VAS score at lateral chest wall.

Data expressed as mean (range, SD).

* Significance, *p *< 0.05.

**Table 4:** Postoperative dynamic VAS (VAS-D) scoring, shown by a group.

	Conventional (group *N*) (*n* = 24)	Cryo (group C) (*n* = 24)	SAPB (group S) (*n* = 26)	*p* value*
VAS-D-A				
1h	8.38 (7–10, 0.824)	7.17 (4–9, 1.204)	5.77 (4–8, 1.520)	<0.001
3h	8.04 (6–10, 1.042)	6.92 (4–8, 0.974)	5.04 (3–7, 1.148)	<0.001
6 h	8.17 (6–10, 1.049)	6.71 (3–9, 1.628)	4.96 (2–7, 1.280)	0.004
12 h	7.83 (5–10, 1.239)	6.17 (4–8, 1.523)	4.50 (3–6, 0.812)	<0.001
24 h	7.75 (4–10, 1.391)	6.13 (2–8, 1.154)	3.96 (1–5, 1.216)	<0.001
48 h	6.75 (3–10, 1.539)	5.07 (2–7, 1.846)	3.77 (2–6, 1.070)	<0.001
72 h	5.88 (3–10, 1.650)	3.92 (2–7, 1.412)	3.62 (1–5, 1.061)	0.003
VAS-D-L				
1 h	7.96 (6–10, 1.16)	6.88 (4–9, 1.42)	5.08 (3–8, 1.35)	<0.001
3 h	7.58 (5–9, 0.97)	6.62 (4–8, 1.17)	4.31 (2–7, 1.29)	<0.001
6 h	7.25 (4–10, 1.73)	6.04 (2–9, 1.90)	4.0 (1–7, 1.38)	0.003
12 h	6.91 (4–9, 1.41)	4.75 (2–8, 1.80)	3.65 (2–6, 1.13)	<0.001
24 h	6.65 (4–10, 1.47)	4.75 (2–8, 1.52)	3.35 (1–6, 1.35)	<0.001
48 h	5.96 (2–10, 1.78)	3.67 (2–7, 1.52)	3.27 (1–5, 1.08)	<0.001
72 h	5.08 (2–10, 1.93)	3.16 (2–6, 1.49)	3.15 (1–5, 1.15)	<0.001

Cryo, cryoanalgesia.

Data expressed as mean (range, SD).

* Significance, *p *< 0.05.

**Figure 5:**
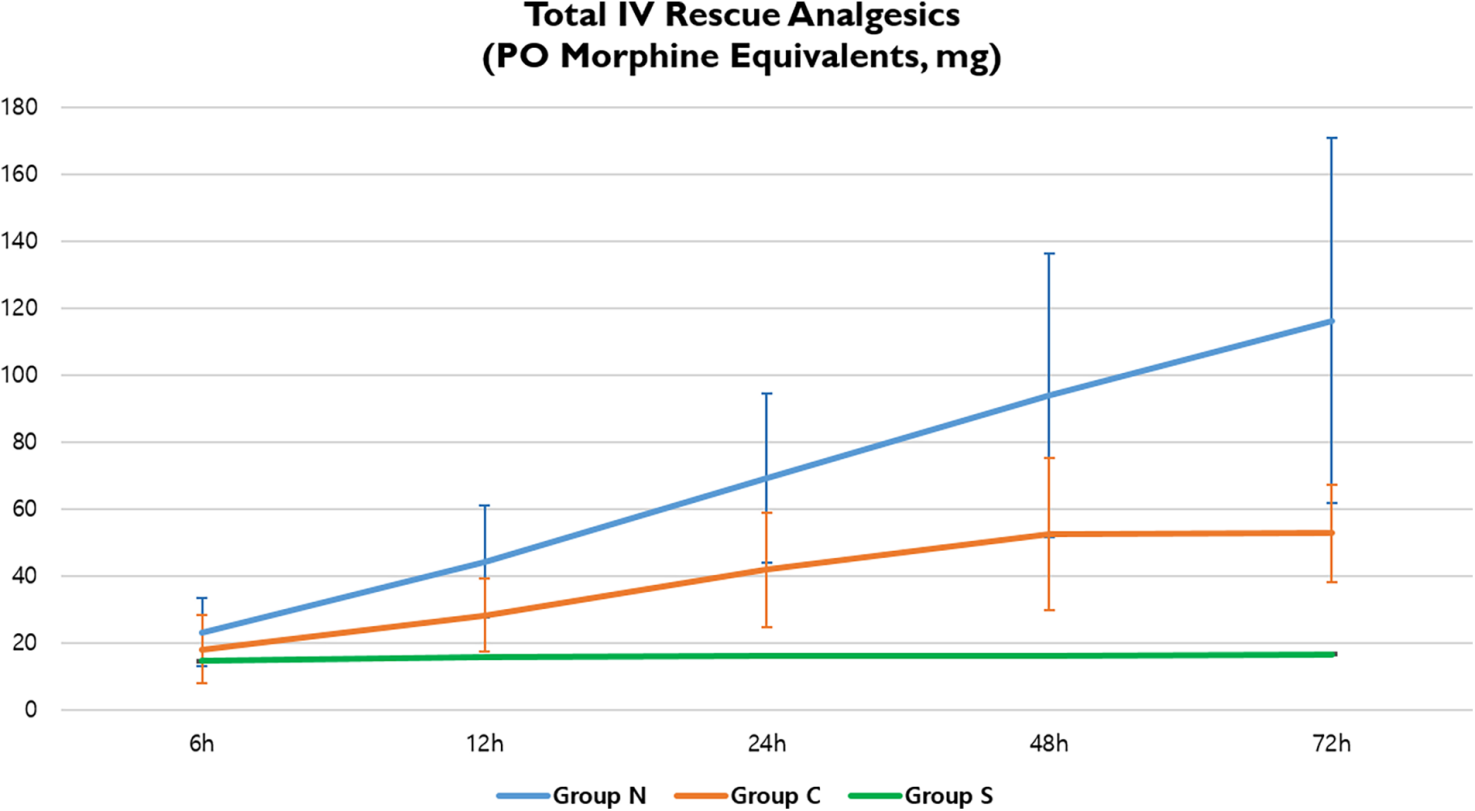
Total IV rescue analgesic consumption in three groups. IV, intravenous; PO, postoperative.

The total IV analgesic consumption in each group administered for breakthrough pain after surgical repair was converted to oral MME.; fentanyl, pethidine, and ketorolac served as IV rescue analgesics. Overall, there were significant differences in total analgesic consumption between the three groups; Group S showed significantly lower MME at 72 h postoperatively (Group *N*, 116.16; Group C, 52.75; Group S, 16.61; *p* < 0.001) (Table 5, Figure 5).

**Table 5:** Total postoperative IV rescue analgesic consumption by a group.

Total IV rescue analgesics, mg^a^	Conventional (group *N*) (*n* = 24)	Cryo (group C) (*n* = 24)	SAPB (group S) (*n* = 26)	*p* value*
6 h	23.18 (10.09)	18.08 (10.28)	14.63 (3.59)	0.003
12 h	44.24 (16.04)	28.24 (10.91)	15.62 (4.15)	<0.001
24 h	69.21 (25.27)	41.89 (17.06)	16.01 (4.34)	<0.001
48 h	93.84 (42.26)	52.50 (22.86)	16.24 (4.35)	0.004
72 h	116.16 (54.55)	52.75 (14.61)	16.61 (4.96)	<0.001

IV, intravenous; MOD, morphine oral-equivalent dose; Cryo, cryoanalgesia; SD, standard deviation.

Data expressed as mean (SD).

* Significance, *p < *0.05.

^a^ Calculated as MOD.

Owing to the nerve block time requirements, the mean operative time in Group C (159.42 min) was significantly longer than that in Group *N* (125.96 min). However, Group S had the shortest operative time, even when a nerve block was performed (110.96 min; *p* = 0.001). The mean cryoanalgesia time for Group C was 30.33 min and the SAPB time for Group S was 17.77 min (*p* < 0.001).

The LOS was not significantly different among the three groups. In Group S, patients stayed for 4.61 days on average, compared with 5.22 and 4.83 days in Groups N and C, respectively (*p* = 0.057). The postoperative complications were not significantly different among the three groups. Four patients in Groups S, C, and N developed postoperative pneumothorax (*p* = 0.85), which required no intervention and resolved spontaneously. One patient in Group *N* had a postoperative wound seroma that was treated with antibiotics (*p* = 0.36). One patient in Group S had postoperative pneumonia, which was treated with antibiotics (*p* = 0.40), and one in Group S had postoperative thoracic outlet syndrome that required no intervention and resolved spontaneously (*p* = 0.45; Table 6). There were no catheter related complications, such as infection, dislocation and obstruction.

**Table 6:** Postoperative outcomes by a group.

	Conventional (group *N*) (*n* = 24)	Cryo (group C) (*n* = 24)	SAPB (group S) (*n* = 26)	*p* value*
Length of stay, days	5.22 (4–8, 1.41)	4.83 (4–8, 1.05)	4.61 (4–7, 0.94)	0.057
Complications				
Pneumothorax	4 (16.67)	4 (16.67)	4 (15.38)	0.845
Wound infection	1 (4.1)	0	0	0.358
Pneumonia	0	0	1 (3.8)	0.403
Thoracic outlet syndrome (TOS)	0	0	1 (3.8)	0.452
Bar dislocation	0	0	0	1.000
Re-operation	0	0	0	1.000

Cryo, cryoanalgesia;.

Data expressed as as mean (range, SD) or *n* (%).

* Significance, *p *< 0.05.

## Discussion

Postoperative pain after PE repair remains challenging. Despite recent advances in surgical techniques and postoperative management, pain after PE repair remains a challenge. Conventionally, IV-PCA has been well established as the first line of postoperative pain control, and on-demand bolus shots of opioids or nonsteroidal analgesics have been used (26). Additionally, local intercostal nerve blocks and paravertebral blocks were also used (26, 27).

Thoracic epidural analgesia is considered one of the most effective methods for pain management in adults undergoing thoracic procedures; however, complications may range from minor catheter issues (kinking or dislodgement) to serious neurological consequences (8, 28). When considering the efficacy or problems related to the modalities of pain management, current pain control methods remain suboptimal.

Recently, cryoanalgesia has been investigated and proven to be relatively superior to conventional pain control methods (10, 12, 29). The cryoprobe is rapidly cooled to the target temperature and applied directly to the targeted intercostal nerve, inducing axonotmesis and reversible transient axonal disruption, which allows for several weeks to months of analgesia (28–35). However, this requires invasive and time-consuming procedures, including single-lung ventilation with double-lumen endotracheal intubation, videoscopy-assisted intrathoracic procedures, and expensive cryo equipment (34, 35). Conversely, SAPB is a new regional block technique for obtaining thoracic local anesthetic analgesia between T2 and T9 (23, 24). SAPB was originally proposed for breast surgery; however, its application has been extended and is highlighted in thoracic surgery, especially in video-assisted thoracic surgery (23, 29). The nerve block can cover the anterior and lateral chest walls, which is the operative field for pectus repair. SAPB involves blocking the intercostal nerve branch with local anesthetic infusion through an extrathoracic catheter. Catheter placement can be conveniently performed using a sonography-guided subcutaneous approach at the operating table after repair.

Bilateral single-injection SAPB in patients undergoing minimally invasive repair of the pectus excavatum decreases pain and opioid consumption compared to those associated with IV-PCA alone during the early postoperative period (14, 31).

However, in our study, we tested the efficacy of continuous SAPB for postoperative pain control and compared the results with those of cryointercostal ablation. For meticulous evaluation of SAPB, we measured the VAS scores in the resting and dynamic states and at dual locations (anterior and lateral chest wall) and identified significant comparative reductions in postoperative resting and dynamic, dual locations (anterior and lateral chest wall).

Assuming a VAS score <4 (mild pain) as the target level for tolerable pain, SAPB improved pain control after PE repair, lowering VAS scores (VAS-R and VAS-D) to this level at 3–24 h postoperatively, whereas cryoanalgesia only reached the level at 48–72 h postoperatively.

The average block time for cryoanalgesia (mean, 30.33 min) exceeded that for SAPB (mean, 17.77 min). Theoretically, VATS intercostal nerve cryoablation requires a minimum of 20 min, as 2 min of each intercostal nerve at the bilateral T4–T7 level is required for the procedure. In contrast, SAPB catheterization is a subcutaneous approach with bedside sonographic guidance.

It is clear that cryoanalgesia added extra time to the procedure due to the need for nerve freezing at bilateral multiple levels. In this study, the reason for the shorter operation time in Group *N* compared to Group S, which requires a block time, is not apparent. However, we believe that the duration of the procedure can be influenced by various factors. In our clinical practice, it often involves additional time for reshaping the pectus bars or, in some instances, modifying the bar placement strategy when it is considered unsuitable for optimal repair.

Other studies have reported significant reductions in LOS and opioid consumption with cryoanalgesia compared to those associated with conventional pain management alone (including thoracic epidural analgesia) (28, 32, 33). However, in our previous study, we did not find total IV rescue analgesics, and LOS was significantly reduced after cryoanalgesia (13).

We are very hopeful of using SAPB for pain management because it is economic, less invasive, and technically straightforward. Moreover, as SAPB takes effect immediately on its infusion, while cryoanalgesia has some latency of the effect, we can handle severe pain upon awakening of the patient from general anesthesia. The pain is most intense during right after the surgery but gradually decreases and becomes bearable as the patient recovers. Rarely we observed persistent pain beyond a few weeks after the operation. Hence, our focus was on relieving the acute pain during the immediate postoperative period, from 6 to 48 h. In this study, we aimed to confirm the effectiveness of pain control during this critical time. The local anesthetic infusion takes effect immediately upon administration, precisely when it is needed most. Although its effect is temporary and only lasts during the infusion, we did not observe any continued, significant pain after discharge or in the subsequent recovery period. Based on our findings, SAPB could be a better option than using cryoanalgesia or IV-PCA alone for controlling acute pain in the early postoperative period, and it has the potential to replace the costly and invasive cryoanalgesia technique.

To our knowledge, this is the first study to investigate the efficacy of continuous SAPB in postoperative pain management by comparing the cryoanalgesic and conventional groups.

However, this study had several limitations. First, the sample size examined was small and the duration was relatively short. Second, the study was retrospective in nature. Third, additional information, such as a longer follow-up outcome, is necessary. Our study was focused on immediate postoperative periods (up to 72 h), it has limitation on evaluation of the patient's longer-term condition after discharge. Fourth, since the preoperative Haller index varied between the three groups, this may have influenced postoperative pain outcomes. Fifth, the different bar patterns of each group may have affected postoperative outcomes. Due to our repair policy shifted towards remodeling the entire anterior chest wall in recent cases, predominantly utilizing the XI fashion approach, which includes cross bars and an upper horizontal bar with three bars in total. While this approach may appear more complex due to the use of three bars (two for cross and three for XI), we hypothesize that it may result in less postoperative pain when combined with SAPB. Sixth, We did not take additional steps, such as injecting a local anesthetic into the intercostal nerves along with cryoablation, which could have served to mitigate the delayed effect of cryo alone. Seventh, We did not do preoperative percutenaous application of ultrasound guided cryoablation due to resource limitations, despite its demonstrated benefits in previous research. Nevertheless, it's worth recognizing that distinct patterns of pectus bars could have influenced postoperative outcomes in each group.

To confirm the validity of these preliminary findings and to establish SAPB as the standard method of pain management after pectus excavatum repair, additional prospective research is required.

## Conclusions

After PE repair, continuous SAPB improved postoperative pain control in both resting and dynamic states. Moreover, the pain intensity reduced to a mild level (VAS score, 0–4 points) immediately postoperatively, and the total IV rescue analgesic consumption diminished. Our study suggests that SAPB could be more effective than the conventional procedure and even cryoanalgesia for immediate postoperative pain relief. However, our results are confined to the initial 72 h post-surgery, specifically within the in-hospital postoperative period. Further study is required to prove the long term efficacy of pain control of SAPB after PE repair.

## Data Availability

The original contributions presented in the study are included in the article/Supplementary Material, further inquiries can be directed to the corresponding authors.
